# Outcomes of Scleral Buckling in Paediatric Rhegmatogenous Retinal Detachment: The Manchester Buckle Study

**DOI:** 10.3390/jcm14165874

**Published:** 2025-08-20

**Authors:** Peter Kiraly, Myrta Lippera, Ritu Agarwal, Tsveta Ivanova, George Moussa, Felipe Dhawahir-Scala, Niall Patton, George Turner, Stephen Charles, Assad Jalil, Kirti Jasani

**Affiliations:** 1 Manchester Royal Eye Hospital, Manchester University Hospitals NHS Foundation Trust, Manchester M13 9WL, UK; 2 Oxford Eye Hospital, Oxford University Hospitals NHS Foundation Trust, Oxford OX3 9DU, UK; 3 Nuffield Laboratory of Ophthalmology, University of Oxford, Oxford OX3 9DU, UK; 4 Department of Ophthalmology, Humanitas Mater Domini, 21053 Castellanza, Italy; 5 Barnet Hospital, Royal Free London NHS Foundation Trust, Wellhouse Lane, Barnet EN5 3DJ, UK

**Keywords:** paediatric rhegmatogenous retinal detachment, scleral buckling, episcleral surgery, paediatric vitreoretinal surgery

## Abstract

**Objectives**: To describe the anatomical and functional outcomes of paediatric rhegmatogenous retinal detachment (RRD) managed primarily with scleral buckle and to identify factors predicting single-surgery anatomical success (SSAS) and postoperative best-recorded visual acuity (BRVA). **Methods**: A retrospective review was conducted of 49 patients (≤18 years) who underwent primary scleral buckle for RRD between 2008 and 2023 at the Manchester Royal Eye Hospital. Data on patient and RRD characteristics, ocular comorbidities, surgical technique, complications, and postoperative outcomes were collected. SSAS, final anatomical success, and BRVA were assessed. **Results**: The mean age at surgery was 12 ± 3 years, with macula-off detachment in 57% (28/49). SSAS after scleral buckle surgery was achieved in 71% (35/49). At the second surgery, 13 out of 14 patients underwent vitrectomy, and one patient had repeat scleral buckling. The final anatomical success rate was achieved in 96% (47/49). On multivariable analysis, older age independently predicted higher odds of SSAS (odds ratio [OR] 1.41, 95% confidence interval [CI] 1.05–1.91, *p* = 0.023), whereas macula status, drainage, and trauma were not independent predictors. In a multivariable linear model for postoperative BRVA (logMAR), older age was associated with better BRVA (B = −0.162, 95% CI −0.244 to −0.080, *p* < 0.001), and macula-off status with worse BRVA (B = 0.520, 95% CI 0.022 to 1.018, *p* = 0.041); drainage and trauma were not significant. **Conclusions**: Primary scleral buckle, with secondary vitrectomy if needed, is effective for paediatric RRD, yielding a 71% SSAS and 96% final anatomical success. Older age was independently associated with higher SSAS and better postoperative BRVA, while macula-off presentation was associated with worse postoperative BRVA.

## 1. Introduction

Paediatric rhegmatogenous retinal detachment (RRD) accounts for 3.2–5.6% of all retinal detachments and is commonly associated with ocular trauma, myopia, and congenital or developmental abnormalities (e.g., familial exudative vitreoretinopathy [FEVR], Marfan syndrome, Stickler syndrome, and retinopathy of prematurity) [1,2]. In contrast to adult RRD, paediatric RRD is commonly linked with congenital or developmental abnormalities, accounting for 35–56% of all paediatric RRDs in Western populations and 12–17% in East Asia [3]. Associations with these abnormalities are even more pronounced in children younger than ten years [3]. Compared to adult RRD, paediatric RRD typically presents with worse initial VA, has a more chronic course, and exhibits a higher rate of macula-off detachment and proliferative vitreoretinopathy (PVR) [4]. Immature cognition in children leads to delayed recognition of acute RRD, with only 40–70% of children reporting vision symptoms at the time of presentation [3]. In the paediatric population with RRD, the vitreous typically remains largely non-syneretic. This differs significantly from adults, where vitreous syneresis (liquefaction) and associated tractional forces facilitate the accumulation of subretinal fluid [2]. Consequently, posterior vitreous detachment (PVD) is rarely seen in paediatric RRD due to the strong vitreoretinal adhesion [2]. In contrast, in adults, PVD and tractional retinal tears are responsible for over 85% of RRD cases [5]. A study comparing scleral buckle versus vitrectomy in non-PVD RRD showed similar anatomical outcomes between both groups, and lower rate of cataract and better visual acuity in the scleral buckle group. Therefore, scleral buckling is the preferred method of RRD repair in the paediatric population, with a reported single-surgery anatomical success (SSAS) of 61–73% using either segmental or encircling explants [6].

In our study, we aimed to describe patient and RRD characteristics, surgical details, and intraoperative findings; assess the postoperative course and complications and anatomical and functional outcomes; and identify predictive factors for SSAS and for postoperative best-recorded visual acuity (BRVA) from a single large tertiary institution.

## 2. Materials and Methods

A retrospective analysis of paediatric patients (age < 18 years) who had scleral buckle surgery at the Manchester Royal Eye Hospital (MREH) from 2008 to 2023 was performed. This study adhered to the tenets of the Declaration of Helsinki, and because all surgeries and investigations were part of routine clinical care, ethical approval was not deemed necessary. Data was obtained using an electronic database search at MREH, gathering all paediatric patients who had scleral buckling as the primary surgery for RRD. Patients with missing/incorrect notes, revisional surgeries, concurrent vitrectomies, and adult patients were excluded.

A retrospective analysis was undertaken to collect demographic information, morphological and functional preoperative characteristics, details of operative management, intraoperative complications, postoperative BRVA, and the SSAS rate. Demographic data comprised sex and age at the time of surgery. Morphological data encompassed laterality, macular status (macula-on or macula-off), and RRD type (dialysis or retinal break related). For ocular comorbidities, high myopia was defined as an axial length greater than 26 mm or a refractive error exceeding −6 diopters, and any recent significant trauma to the affected eye was noted. BRVA was converted to logMAR for statistical analysis. BRVA was measured by an optometrist under standardised conditions, using optimal refractive correction or a pinhole as appropriate. Postoperative BRVA was assessed at the 2-month follow-up visit after primary scleral buckling. We defined SASS as complete retinal reattachment after the initial scleral buckle procedure, without needing any further surgical intervention to address the retinal detachment. Final anatomical success was defined as complete retinal reattachment following the last vitreoretinal procedure, achieved without the use of silicone oil tamponade. Patients with poor fundal view (corneal opacities, vitreous haemorrhage, etc.), PVR C, posterior retinal breaks, familial exudative vitreoretinopathy, or significant vitreous traction were excluded from this study and underwent primary PPV.

Surgery comprised retinal cryopexy with cryocoagulation followed by placement of a silicone sponge or tire explant sutured to the sclera using hemi-Halsted mattress sutures. Indirect ophthalmoscopy was used for intraoperative visualisation. The posterior edge of the buckle was positioned at least 1 mm posterior to the most posterior retinal hole. The decision to perform external drainage was at the surgeon’s discretion. External drainage in the area of maximal subretinal fluid was generally undertaken in eyes with extensive fluid and lack of apposition between the retinal hole and the indent. The sclera and choroid were perforated with a suture needle, and immediate pressure adjacent to the entry site facilitated fluid egress and minimised haemorrhage. The type of explant used was left to the surgeon’s preference. Nevertheless, sponges were predominantly used in dialysis RRD, as they provide a higher anterior indent, while tires were more commonly used in atrophic hole RRD. Segmental buckles were used in most patients, as those with poor fundal view were excluded and retinal holes or dialysis could be reliably localised, making encircling bands rarely necessary.

### Statistical Analyses

Analyses were performed in SPSS (Version 20.0; IBM Corporation, Armonk, NY, USA). Univariate comparisons used Student’s t-test or Mann–Whitney U for continuous data and χ^2^ or Fisher’s exact tests for categorical data; correlations with BRVA used Pearson’s correlation coefficient. Multivariable models assessed independent effects: binary logistic regression for SSAS (1 = success, 0 = failure) and linear regression for postoperative BRVA (logMAR), entering age (years), macula-off (yes = 1), external drainage (yes = 1), and trauma (yes = 1) simultaneously. We report adjusted odds ratios (ORs) or unstandardised coefficients (B) with 95% confidence intervals (CIs) (plus adjusted R^2^ for the linear model); logistic fit/calibration were summarised by the likelihood-ratio χ^2^ and Hosmer–Lemeshow tests. All *p*-values are two-sided, with significance at *p* < 0.05.

## 3. Results

### 3.1. Methods and Patient Characteristics

Our study included 49 paediatric patients with a mean age at surgery of 12 ± 3 (range 1–15) years and a mean follow-up duration of 3.6 ± 3.5 years. Among these patients, 34 (69%) were male and 15 (31%) were female. The right eye was involved in 23 cases (47%), while the left eye was affected in 26 cases (53%). Twenty-one patients (43%) presented with macula-on RRD, whereas 28 (57%) had macula-off RRD. Dialysis-related RRDs were observed in 18 patients (37%), while retinal hole RRDs were identified in 31 patients (63%). Regarding other ocular comorbidities, 7 patients (14%) had high myopia, and 10 (20%) had ocular trauma. Additionally, four patients had Stickler syndrome, while one patient each was diagnosed with retinopathy of prematurity, retinitis pigmentosa, microphthalmos, and morning glory disc anomaly. At presentation, the average BRVA was 0.75 ± 0.68 logMAR in all eyes.

### 3.2. Surgical Details and Intraoperative Findings

Scleral buckle procedures were performed by consultants in 36 eyes (73%) and by fellows in 13 eyes (27%). External prang drainage of subretinal fluid was performed in 16 eyes (32%). With regards to scleral explants, tires were used in 28 eyes (57%) and sponges in 21 (43%). A segmental buckle was used in all surgeries, while an additional encircling band was used in two patients. Table 1 presents preoperative characteristics and intraoperative management. In terms of intra-operative complications, small and localised subretinal haemorrhages at the drainage site were observed in two patients who underwent drainage; there were no cases of subretinal haemorrhage involving the macula or intravitreal haemorrhage. One patient required an intravitreal air injection during surgery because of transient hypotony following subretinal fluid drainage.

### 3.3. Postoperative Course and Complications

SSAS was achieved in 35 eyes (71%). Postoperative laser treatment was required in five eyes: in four eyes for retinal tears and in one eye for peripheral retinal ischemia. Two patients developed buckle extrusion, of which one underwent explant removal. Elevated intraocular pressure (IOP) occurred in seven patients (14%), and one of these patients required glaucoma surgery. One patient developed persistent diplopia, which was managed with strabismus surgery.

A second surgery was required in 14 patients: 13 underwent pars plana vitrectomy, and 1 had repeat scleral buckling with cryotherapy. Primary scleral buckling in our patients failed due to missed holes, insufficient indentation, or incorrect buckle placement. The mean interval between the first and second surgeries was 1.6 ± 0.9 months. Final anatomical success was achieved in 47 eyes (96%). Overall, 35 patients (71%) required only one surgery, 6 (12%) required two, 3 (6%) required three, 2 (4%) required four, and 1 (2%) required six.

### 3.4. Predictive Factors for Single-Surgery Anatomical Success Rate

Mean (±SD) age in the successful SASS group was 12.4 ± 2.23 years, compared with 10.1 ± 3.45 years in the unsuccessful SASS group (*p* = 0.04). Among macula-on eyes, the SASS was 86% (18/21), versus 61% (17/28) in macula-off eyes (*p* = 0.06). SASS did not differ significantly when comparing the presence of dialysis (78% vs. 68%; *p* = 0.45), retinal break (68% vs. 78%; *p* = 0.44), high myopia (86% vs. 69%; *p* = 0.37), or trauma (70% vs. 72%; *p* = 0.90). In eyes with external drainage, the SASS was 63%, whereas those without drainage achieved 76% (*p* = 0.33). When comparing explants, eyes treated with sponges had a SASS of 81%, compared with 64% for tires (*p* = 0.20). Surgeon grade was not predictive of SSAS (*p* = 0.08). Table 2 presents univariate predictive factors for SASS.

We fitted a multivariable logistic regression to identify independent predictors of SSAS. Overall model fit was acceptable (likelihood-ratio χ^2^(4) = 9.83, *p* = 0.043) with adequate calibration (Hosmer–Lemeshow *p* = 0.29). Older age independently predicted higher odds of SSAS (OR 1.41, 95% CI 1.05–1.91, *p* = 0.023), while macula status, drainage, and trauma were not statistically significant after adjustment (Table 3).

### 3.5. Predictive Factors for Postoperative Best Recorded Visual Acuity

The correlation between age at surgery and postoperative BRVA was non-significant (Pearson r ≈ −0.15; *p* = 0.30). Among macula-on eyes (n = 19), the mean BRVA was 0.36 ± 0.25, compared with 0.97 ± 0.95 in macula-off eyes (n = 27; *p* < 0.01). Postoperative BRVA did not differ significantly by type of RRD (dialysis vs. breaks; *p* = 0.30). Also, BRVA did not differ significantly with the presence of high myopia (*p* = 0.21) and trauma (*p* = 0.37). In eyes without external drainage (n = 32), the mean BRVA was 0.66 ± 0.55, compared with 0.86 ± 0.60 in eyes with drainage (n = 14; *p* = 0.13). Finally, when comparing explant types, eyes treated with tires (n = 26) had a mean BRVA of 0.92 ± 0.70, compared with 0.45 ± 0.35 in eyes treated with sponges (n = 20; *p* = 0.13). Table 4 presents univariate predictive factors for postoperative BRVA.

We fitted a multivariable linear regression to identify independent predictors of postoperative BRVA (logMAR). The model explained 31% of the variance (Adjusted R^2^ = 0.31); overall F(4,41) = 6.01, *p* < 0.001. Older age was associated with better BRVA (B = −0.162, 95% CI −0.244 to −0.080, *p* < 0.001), while macula-off status was associated with worse BRVA (B = 0.520, 95% CI 0.022 to 1.018, *p* = 0.041). Drainage and trauma were not statistically significant after adjustment (Table 5).

## 4. Discussion

Our study demonstrated a 71% SSAS and a 96% final anatomical success rate in pediatric patients treated with primary scleral buckle, followed by pars plana vitrectomy when necessary. Macula-on RRDs were associated with better anatomical outcomes and superior postoperative BRVA, while older age at presentation also correlated with more favorable anatomical results.

In our study, 69% of the patients were male, which is slightly lower than previous studies reporting a 70–79.5% [2,7,8] male predominance among paediatric RRD patients but higher than the 51–64% [2,9] observed in adult RRD patients. Studies reported that this difference could be attributed to the higher incidence of ocular trauma in boys compared to girls [2,6,7,8]. We also found that 57% of our patients presented with macula-off RRD, placing our findings at the lower end of the range reported in pediatric literature (59–94%) [2,6,7,8] yet still higher than the 45% typically observed in adults with RRD [2]. In general, macula-off RRDs are more frequent in children, especially at a younger age, likely due to delays in diagnosis and treatment due to difficulties in recognising or communicating visual symptoms, leading to later presentation and more advanced disease at the time of diagnosis [2]. Nonetheless, the average age at presentation (12 years) in our study is similar to other paediatric studies (10.8–13.1 years) [2,6,7,8] and could not explain the lower macula-off RRDs in our results. Retinal dialysis was present in 37% of patients, a rate lower than the previously reported 54% in paediatric RRD [6] and significantly higher than in all RRD (6–17%) [10]. Retinal dialysis RRD is associated with trauma in 61.4% of cases [10]. In our study, trauma was present in 20% of eyes, which is lower than previously reported in paediatric RRD (33–43%) [2,6,7]. In pediatric RRD, the proportion of trauma-related cases may be underestimated, as obtaining a reliable history can be challenging, particularly in younger children. In our cohort, only 14% of patients had high myopia (>6 dioptres). This percentage is slightly lower than the 25% reported in a UK-based study [6] and markedly lower than the 37.5% of children with myopia exceeding 4 dioptres reported in a Taiwan-based study [7], which may reflect the greater prevalence of myopia in East Asia compared to our cohort. In our cohort, 16% of eyes had congenital or developmental anomalies, which is significantly lower than the rates reported in previous paediatric RRD studies [4,8]. However, our study includes only patients who underwent scleral buckling. Many patients with congenital or developmental anomalies develop complex RRD, for which PPV is the preferred surgical approach, and therefore were not included in our sample. The exclusion of more complex RRD cases may have resulted in better anatomical and functional outcomes, which could limit the generalisability of our findings.

External drainage was performed in 32% of cases, which is comparable to the drainage rate for scleral buckle surgery in adults at the MREH (30%; unpublished data). In another paediatric buckle study, the drainage rate was 37.5% [6], while previously published studies of adult patients report drainage rates ranging from 50% to 84% [11,12]. In 57% of cases, tires were used, while in 43% of eyes, sponges were used, a distribution similar to that previously reported in adult scleral buckle surgery [13]. In 96% of our patients, only a segmental explant was used. Previous studies used either predominantly segmental explants or encircling bands, with success rates similar to ours [3,6,7,14,15,16]. Drainage resulted in two small, localised peripheral subretinal haemorrhages and one case of transient hypotony, which resolved following an intravitreal air injection. Consequently, there were no intraoperative complications that led to a worsening of visual function.

The SSAS in our study was 71%, and final anatomical success was achieved in 96% of eyes. Our SSAS was very similar to a previous study where primary scleral buckling was performed for paediatric RRD, which reported an SSAS of 73% and a final anatomical success rate of 94% [6]. However, previous paediatric RRD studies have reported lower success rates, with SSAS ranging from 50–80% and final anatomic success rates of 70–80% in most cohorts [3]. Lower anatomical success rates in these studies could be attributed to the inclusion of younger patients with more severe RRD requiring vitrectomy [3], while our study included less severe RRD suitable for a primary buckle. The SSAS in adult patients undergoing scleral buckle as the primary procedure is significantly higher, ranging from 84 to 91% [9,11,17]. The rates of intraoperative and postoperative complications were similar in our paediatric and adult cohorts (unpublished data). In our cohort, 13 of 14 patients with unsuccessful primary retinal reattachment underwent vitrectomy, resulting in a final anatomical success rate of 96%. Although Errera et al. reported about half of their failures with buckle and half with vitrectomy, their final anatomical success rate (94%) was comparable to ours [6].

Regarding predictive factors for SSAS, older age was associated with improved outcomes. This finding was also reported by Wang et al. [18], who observed the poorest anatomical and functional outcomes in children younger than eleven years, potentially due to more complex retinal detachments or associated pathology in this younger age group. Additionally, although not statistically significant, SSAS tended to be worse in children with macula-off RRD, possibly reflecting the greater severity or extent of disease in these cases. There were no differences in SSAS based on the type of RRD (dialysis or retinal break) or ocular comorbidities (high myopia, trauma). On the other hand, other studies have identified trauma as a risk factor for poor outcomes, alongside congenital abnormalities, prior surgery, PVR, and giant retinal tears [3]. Most children with these RRD characteristics were not included in our study because they underwent primary vitrectomy with additional retinal explant if needed. In our study, patients who had external drainage showed an SSAS rate of 63% compared with 76% in those without drainage; the difference was not statistically significant and aligns with studies reporting similar SSAS in adults with and without drainage [11,12]. The slightly lower SSAS rate observed in the drainage group may be linked to more advanced or complex cases of RRD, with an increased amount of subretinal fluid. Our results also showed a better SSAS rate with sponges (81%) than with tires (64%), although this was not statistically significant—likely due to the relatively small sample size. Out of predictive factors for postoperative BRVA, macula-on RRD was associated with better BRVA outcomes in our study. Postoperative BRVA was the only functional outcome routinely recorded. Owing to the retrospective design, other functional outcomes (e.g., binocular vision, quality-of-life measures) were not collected and could not be analyzed; these should be evaluated in future prospective studies.

Primary scleral buckle offers several advantages over primary vitrectomy in children [3]. Firstly, the strong adherence of the posterior hyaloid to the retina makes PVD induction challenging when performing vitrectomy. Secondly, the absence of internal tamponade, such as long-acting gas or silicone oil, reduces the risk of the development of amblyopia in younger children. And finally, patients are much less likely to develop cataracts after a scleral buckle compared to a vitrectomy. This is especially pertinent since paediatric cataracts have significantly greater morbidity with their amblyogenic potential and loss of accommodation following surgery [3].

Our study confirms prior reports of good anatomical outcomes after primary scleral buckle [6]. To our knowledge, we are the first to explore predictors of postoperative BRVA and to evaluate the effects of external drainage and explant type on anatomical outcomes. Our findings show that segmental buckling alone yields favorable anatomical and functional outcomes; therefore, an encircling buckle may not be required. The limitations of our study include its retrospective design, limited available data, and uneven follow-up. In addition, the subgroup analyses were small and underpowered, which could result in non-significant findings due to type II error. Furthermore, no correction for multiple hypothesis testing was applied, which could increase the risk of type I error. Nevertheless, we confirmed the univariate results with multivariable logistic regression.

## 5. Conclusions

In conclusion, our study demonstrated good anatomical outcomes in paediatric RRD treated with a primary scleral buckle, with secondary vitrectomy if needed. This approach achieved a SSAS success rate of 71% and a final anatomical success rate of 96%. Older age and macula-on RRD were associated with a higher SSAS rate, whilst macula-on RRD was associated with better postoperative BRVA. These findings support scleral buckle as a valuable first-line option in children, particularly due to better functional outcomes associated with reduced development of cataracts in paediatric patients.

## Figures and Tables

**Table 1:** Preoperative characteristics and intraoperative management.

Parameter	Value
Number of patients	49
Follow-up duration (mean [years] ± SD)	3.6 ± 3.5
Sex (male/female)	34 (69%)/15 (31%)
Mean age at surgery (mean [years] ± SD)	12 ± 3
Operated eye (right/left)	23 (47%)/26 (53%)
Macula-on/-off	21 (43%)/28 (57%)
Type of detachment (dialysis/break)	18 (37%)/31 (63%)
High myopia	7 (14%)
Ocular trauma	10 (20%)
Preoperative BRVA ^1^ (mean [logMAR] ± SD)	0.75 ± 0.68
Surgeon (consultant/fellow)	36 (73%)/13 (27%)
External drainage performed	16 (32%)
Explant Type (tire/sponge)	28 (57%)/21 (43%)

^1^ BRVA: Best recorded visual acuity.

**Table 2:** Univariate predictive factors for single-surgery anatomical success rate.

Parameter	Comparison (No. of Eyes = Success/Total, %)	*p*-Value
Age (years)	Unsuccessful (n = 14): 10.1 ± 3.45 Successful (n = 35): 12.4 ± 2.23	0.04
Macula	On (n = 21): 18/21 (86%) Off (n = 28): 17/28 (61%)	0.06
Dialysis	Present (n = 18): 14/18 (78%) Absent (n = 31): 21/31 (68%)	0.45
Retinal break	Yes (n = 31): 21/31 (68%) No (n = 18): 14/18 (78%)	0.44
High Myopia	Yes (n = 7): 6/7 (86%) No (n = 42): 29/42 (69%)	0.37
Trauma	Yes (n = 10): 7/10 (70%) No (n = 39): 28/39 (72%)	0.90
External Drainage	Yes (n = 16): 10/16 (63%) No (n = 33): 25/33 (76%)	0.33
Explant Type	Sponges (n = 21): 17/21 (81%) Tires (n = 28): 18/28 (64%)	0.20
Surgeon Grade	Consultant (n = 36): 23/36 (64%) Fellow (n = 13): 12/13 (92%)	0.08

**Table 3:** Independent predictors of single-surgery anatomical success (SSAS)—logistic regression.

Predictor	Adjusted OR (95% CI)	*p*-Value
Age (years)	1.41 (1.05–1.91)	0.023
Macula (1 = off vs. 0 = on)	0.38 (0.08–1.81)	0.222
Drainage (1 = yes vs. 0 = no)	0.50 (0.11–2.18)	0.353
Trauma (1 = yes vs. 0 = no)	0.70 (0.12–4.26)	0.701

Outcome coded 1 = success, 0 = failure; covariates entered together. Model notes: −2LL = 48.80; Cox–Snell R^2^ = 0.182; Nagelkerke R^2^ = 0.260; Hosmer–Lemeshow χ^2^(7) = 8.47, *p* = 0.29. OR = odds ratio; CI = confidence interval.

**Table 4:** Univariate predictive factors for postoperative best recorded visual acuity.

Parameter	Subgroup Findings	*p*-Value
Macula	Macula-on (n = 19): 0.36 ± 0.25 Macula-off (n = 27): 0.97 ± 0.95	<0.01
Dialysis	No (n = 28): 0.91 ± 0.70 Yes (n = 18): 0.41 ± 0.30	0.30
Retinal Break	No (n = 18): 0.41 ± 0.30 Yes (n = 28): 0.91 ± 0.70	0.30
High Myopia	No (n = 40): 0.77 ± 0.60 Yes (n = 6): 0.34 ± 0.25	0.21
Trauma	No (n = 35): 0.84 ± 0.70 Yes (n = 11): 0.33 ± 0.30	0.37
External Drainage	No (n = 32): 0.66 ± 0.55 Yes (n = 14): 0.86 ± 0.60	0.13
Explant Type	Tires (n = 26): 0.87 ± 1.09 Sponges (n = 20): 0.48 ± 0.57	0.13

**Table 5:** Independent predictors of postoperative BRVA—linear regression.

Predictor	B (95% CI)	*p*-Value
Age (years)	−0.162 (−0.244 to −0.080)	<0.001
Macula (1 = off vs. 0 = on)	0.520 (0.022 to 1.018)	0.041
Drainage (1 = yes vs. 0 = no)	0.037 (−0.474 to 0.549)	0.883
Trauma (1 = yes vs. 0 = no)	−0.301 (−0.888 to 0.286)	0.306

Dependent variable: postoperative BRVA (logMAR). Model notes: R = 0.608, R^2^ = 0.370, Adjusted R^2^ = 0.308; ANOVA F(4,41) = 6.01, *p* < 0.001. Assumptions: residual diagnostics unremarkable; collinearity acceptable (VIFs ≈ 1). CI = confidence interval.

## Data Availability

Data sets generated during the current study are available from the corresponding author on reasonable request.
